# Changes in soil carbon fractions and enzyme activities under different vegetation types of the northern Loess Plateau

**DOI:** 10.1002/ece3.6852

**Published:** 2020-09-29

**Authors:** Haiyan Wang, Jiangqi Wu, Guang Li, Lijuan Yan

**Affiliations:** ^1^ College of Forestry Gansu Agricultural University Lanzhou China; ^2^ College of Agriculture Gansu Agricultural University Lanzhou China

**Keywords:** enzyme activities, Loess Plateau, soil organic carbon components, vegetation types

## Abstract

Knowledge of the soil organic carbon components and enzyme activities during long‐term natural vegetation restoration is essential for managing the restoration of vegetation. In this study, the variations of soil organic carbon components (i.e., soil organic carbon (SOC), microbial biomass carbon (MBC), easily oxidized carbon (EOC), particulate organic carbon (POC)) and enzyme activities (i.e., amylase, catalase, urease, and sucrase) were measured in four vegetation types: control (grasslands, GL), forest (*Xanthoceras sorbifolia*, XS), and shrublands (*Hippophae rhamnoides*, HR; *Caragana korshinskii*, CK). We found that vegetation types significantly affect soil organic carbon components and enzyme activities. The SOC content of the XS plot is higher than HR, CK, and GL by 88.43%, 117.09%, and 37.53% at the 0–20 cm layer; the soil SOC content of the XS plot is higher than HR and CK by 27.04% and 26.87%, and lower than GL 12.90% at the 20–40 cm layer. The highest POC and urease were observed in the XS plot at a depth of 0–20 cm, that is, 1.32 g/kg and 98.51 mg/kg, respectively. The highest EOC, amylase, and sucrase were observed in GL at a depth of 0–20 cm, that is, 5.44 g/kg, 39.23, and 607.62 mg/g. On the vertical section of the soil, the SOC fractions and the enzyme activities were greater in the upper layer than in the lower layer for each vegetation type except for MBC and catalase activity. Correlation analysis demonstrated that the SOC and POC content significantly influenced urease and sucrase activities and that MBC significantly influenced catalase activity. These results provide important information about SOC fractions and enzyme activities resulting from vegetation types in the Loess Plateau and also supplement our understanding of soil C sequestration in vegetation restoration.

## Graphical abstract

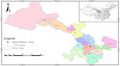

## 1 INTRODUCTION

Soil organic carbon (SOC) can reflect soil health and plays an extremely important role in increasing soil carbon storage, improving soil fertility, and promoting plant growth (Li et al., 2018; Sollins et al., 2007). However, the SOC group consists of subgroups with variable turnover rates, each with a different sensitivity to environmental changes (Guo et al., 2018). Soil active organic carbon typically includes microbial biomass carbon (MBC), easily oxidized carbon (EOC), and particulate organic carbon (POC). While the proportion of soil active organic carbon to soil total organic carbon is low, this ratio can reflect the changes in soil carbon groups due to soil management measures and environmental changes (Jha et al., 2012; Sahoo et al., 2019). The soil active organic carbon is directly involved in the biological and chemical conversion process of soil (Sun et al., 2014), plays a vital role in the cycling of soil nutrient, and stores soil nutrients (Simard et al., 2001). Furthermore, soil active organic carbon is easily affected by plants and microorganisms in a significant way (Chen et al., 2010; Kimura et al., 2004). However, variation in soil active organic carbon contents across different vegetation types is poorly understood.

Soil enzyme activities are involved in the biochemical processes of the soil system and are linked to “plant‐soil enzymes‐soil nutrients” (Araújo et al., 2013; Lino et al., 2015; Nannipieri et al., 2012; da Silva et al., 2012). In particularly, enzyme activities (i.e., amylase, catalase, urease, and sucrase) related to the soil carbon cycle and serve as important indicators of soil fertility. Amylase and sucrase are involved in the conversion of soil carbohydrates and can hydrolyze organic matter into glucose and sucrose for plant growth and microbial activity (Ge et al., 2011; Xie et al., 2017). Urease acts on carbon–nitrogen bonds in organic matter and produces carbon dioxide and water by hydrolyzing ammonia or amino salts, while catalase is related to the redox ability of the soil (Baddam et al., 2016; Nowak et al., 2004). These enzyme activities have an important influence on the carbon cycles in soil ecosystems (Bergstrom et al., 1999; Burns et al., 2013). Previous studies have shown that plants can not only directly influence soil enzyme activities by secreting exogenous enzymes, but also affect the composition and diversity of microbial species by releasing exudate and oxygen into the rhizosphere, which indirectly affect enzyme activity (Singh & Kumar, 2008). Moreover, plants also indirectly mediate enzyme activities in the soil by controlling the volume of aboveground litter (Caravaca et al., 2005). Therefore, these enzyme activities were often chosen to understand the variations in SOC and soil quality (Acosta‐Martínez et al., 2007; Chen et al., 2016).

The Loess Plateau is located in the north‐central China and has one of the highest concentrations of loess on earth, with a total area of 64,000 square kilometers. It also has a high rate of soil erosion and is one of the most ecologically fragile environments in the world, meaning vegetation is important to enhance fertility levels and the soil's ability to hold water (García et al., 1994). Over the past few decades, extensive efforts at restoring the environment have improved the fragile natural ecosystems on the Loess Plateau (Intergovernmental Panel on Climate Change, 2014). Vegetation restoration not only benefits for water preservation and reduction of soil erosion (Ran et al., 2013), but significantly improves the properties and quality of soil (Zhang et al., 2019). Studies have shown that returning farmland to forests not only improves the SOC reserves and quality, but also improves the conversion trend of soil SOC‐related fractions (Deng et al., 2019; Liu et al., 2014; Xun et al., 2010). The vegetation coverage of the Loess Plateau had increased by 28.0% from 1999 to 2013 (Li et al., 2019). Studies have shown that vegetation types can change the SOC content of different soil layers by providing organic carbon to the soil via root turnover and exudation, and leaf litter (Guo et al., 2018; Zhang et al., 2010). For example, SOC stocks in the Loess Plateau increased by 19% in the 0–20 cm surface soil from 1998 to 2006 (Wang et al., 2011), while the SOC stocks in the 0–30 cm soil layer are highly variable under the different vegetation communities (Yimer et al., 2006). Because the litter and root exudates of different vegetation types have a direct impact on soil properties (Gentile et al., 2011; Kooch et al., 2017), such as soil organic carbon fractions (Austin & Ballaré, 2010; de Medeiros et al., 2017) and enzyme activities (Boeddinghaus et al., 2015). Therefore, we used grassland (GL) as a control treatment and selected vegetation from forests (genus *Xanthoceras*, *Xanthoceras sorbifolia*, XS) and shrublands (genus *Hippophae*, *Hippophae rhamnoides,* HR; genus Caragana, *Caragana korshinskii,* CK) on the Loess Plateau to study the distribution characteristics of soil active organic carbon components and soil enzyme activities under different vegetation types. This provided a reference value for the sustainable ecological restoration of the Loess Plateau and subsequent improvement of the soil. We hypothesized that: (a) there are differences among soil organic carbon fractions and enzyme activities in various of different vegetation types due to the change of vegetation types and litter amounts (Liu et al., 2015; de Medeiros et al., 2017), and (b) the SOC components and enzyme activities in the surface layer of all vegetation types were higher than in the lower layer (Zhao et al., 2018).

## 2 MATERIALS AND METHODS

### 2.1 Site description

**FIGURE 1:**
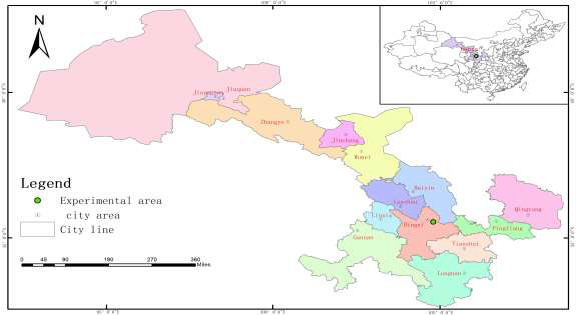
The geographical location map of the study area

The study area (103°52′–105°13′E, 34°26′–35°35′N) is located in the middle of Gansu Province in northwest China (Figure 1) and possesses the hills and gullies typical of the middle of the Loess Plateau. Its average elevation is about 1,947 m. It has a semi‐arid climate and average annual precipitation of about 390.99 mm, which falls mostly from July to September following the harvest season. The soil is a typical loess soil, which is soft and prone to erosion. The action formally launched in 2002, the Conversion of Cropland to Forest and Grassland Program (Wang & Bennett, 2008), and planted vegetation was according to the ratio of 1:2:4 for arbor forest, shrub forest, and grassland. In the early stage of afforestation, the survival rate of returning farmland to forests should be improved by constructing water conservation projects (such as building reservoirs for irrigation) and replanting measures. The major tree species planted during each period of reforestation are *X. sorbifolia*, *C. korshinskii*, and *H. rhamnoides*. *Xanthoceras sorbifolia* (genus Xanthoceras, family Sapindaceae) is a unique woody oil tree to China with a planting area of more than 170,000 ha (Ma et al., 2020). *Caragana korshinskii* (genus Caragana, family Leguminosae) is a suitable shrub species for plantation/afforestation to restore degenerated land (Wang et al., 2019). Its unique stress resistance characteristics make it an important forage, industrial raw material, and afforestation species and have various economic and ecological values (Zhang et al., 2009). *Hippophae rhamnoides* (genus Hippophae, family Elaeagnaceae), also known as sea buckthorn and have the characteristics of drought tolerance, cold tolerance, and barren tolerance. It is one of the few tree species that can grow in the forest zone, forest‐steppe zone, and typical steppe zone at the same time. *Hippophae rhamnoides* berries have been used as traditional medicine for a long time due to their richness in various bioactive ingredients (Sharma et al., 2019; Wei et al., 2019). The composition of dominant species of vegetation and the history of land disturbances in different vegetation types are shown in Table 1.

**TABLE 1:** The basic information of different vegetation types

Vegetation	Main species	Coverage/%	Plants height/m	Disturbance history
XS	*Xanthoceras sorbifolia*, *Agropyron cristatum (Linn.) Gaertn, Bupleurum chinense*）	60	6	Deforested and cultivated before 2002, then abandoned, and artificially recovered to the forest.
HR	*Hippophae rhamnoides*, *Medicago sativa*, *Agropyron cristatum (Linn.) Gaertn*	85	0.6	Deforested and cultivated before the 1990s, abandoned at the end of 2000, and naturally recovered to a shrub community.
CK	*Caragana korshinskii*, *Agropyron cristatum (Linn.) Gaertn*	30	1	
GL	*Agropyron cristatum (Linn.) Gaertn, Artemisia frigida Willd.Sp.Pl*., *Stipa grandis P.Smirn*.	>90	0.2	Abandoned in 1990, natural recovery to a grassland.

Abbreviations: CK, *Caragana korshinskii*; GL, Grassland; HR, *Hippophae rhamnoides*; XS, *Xanthoceras sorbifolia*.

### 2.2 Experimental design and soil sampling

In the experimental site, the GL (Area: 30 × 40 m; 35°34′54″N, 104°37′57″E) was the control group, while XS (Area: 20 × 50 m; 35°35′10″N, 104°37′7″E), CK (Area: 30 × 30 m; 35°34′55″N, 104°38′1″E), and HR (Area: 20 × 30 m; 35°34′45″N, 104°39′1″E) were designated as the three vegetation types (Table 1). Three sample plots (with the size of 8 × 8 m) were randomly selected from each vegetation type for sampling.

In September 2017, the soil sampler (diameter 5 cm) was used to sample layers (0–20, 20–40 cm) according to the diagonal 5‐point method (four points were selected at both ends of an “X,” with one point selected at the intersection). Five soil samples of the same soil layer in each plot were mixed to form one soil sample for a total of 24 soil samples. At the forest plot, soil samples were collected at a distance of approximately 80 cm from trees; while at the HR and CK plots, samples were collected at a distance of approximately 10 cm from shrublands. After removing debris, such as stones and residual roots, the sample was divided into two parts. One part of the fresh soil was stored in a refrigerator at 4°C through a 2 mm soil sieve. Another part of the soil was air‐dried (direct sunlight was avoided on soil samples), placed in a ziplock bag through a 100‐mesh soil sieve, and stored in a cool and ventilated place (storage time not exceeding 1 year).

### 2.3 Soil physical and chemical characteristics

For each selected plot, undisturbed soil samples were collected from two layers: 0–20 cm and 20–40 cm using a cutting ring (Guo et al., 2018). The undisturbed soil samples were weighed and dried in an oven at a uniform temperature of 105°C for 24 hr. The dried soil samples were weighed and the standard formulas were used to calculate the bulk density (BD) and total porosity. The soil sample (1 g, accurate to 0.001 g) was digested with 5 ml of concentrated H_2_SO_4_ at 400°C. When the color of the extracting solution became milky white, heating was stopped and the whole solution was transferred to a 100 ml volumetric flask. 5 ml of the solution was used to measure total nitrogen (TN) with the Semi‐Micro Kjeldahl method (McGill & Fig ueiredo, 1993). 10 ml of the solution was used to determine total phosphorus (TP) with the Mo‐Sb colorimetric method (Lu, 2000).

### 2.4 Soil carbon fractions

SOC was determined by the Walkley–Black dichromate oxidation method (Nelson, 1982), using a mixture of potassium dichromate (K_2_Cr_2_O_7_) and sulfuric acid (H_2_SO_4_) to oxidize the organic matter, after which it was titrated against ferrous sulfate (FeSO_4_). The air‐dried soil sample (0.1 g) was extracted with 7.5 ml of K_2_Cr_2_O_7_ and 7.5 ml of concentrated H_2_SO_4_ at 180°C for 30 min.

MBC was determined on fresh soil samples (sieving < 2 mm) using the chloroform fumigation‐extraction method (Vance et al., 1987). The fumigated soil and non‐fumigated soil (5 g, accurate to 0.001 g) were extracted with 20 ml 0.5 M of K_2_SO_4_ for 30 min on a shaker (180 rpm). 5 ml of supernatant was extracted and titrated according to the method of organic carbon. MBC was calculated as (*C*_fumigated_ − *C*_non‐fumigated_)/0.38 (McLatchey & Reddy, 1998).

POC was determined with necessary modifications based on the method described by Yang et al. (2009). Twenty grams of the soil were dispersed with 100 ml of a 5 g/L sodium hexametaphosphate solution by hand shaking the mixture for 15 min and placing it on a reciprocal shaker (100 rpm) for 16 hr. The dispersed soil sample was then passed through a 53 μm sieve and rinsed thoroughly with distilled water. The remaining material was dried at 50°C, weighed, and finely ground.

EOC was measured using the method of 0.333 M KMnO_4_ oxidation (Blair et al., 1995; Xiao et al., 2015). Briefly, 2 g soil was placed in a 50 ml centrifuge tube and 25 ml of 0.333 M KMnO_4_ solution was added, shaken for 1 hr (180 r/min), and centrifuged for 5 min (5,000 rpm). 1 ml the supernatant was diluted 250 fold and absorbance at 565 nm was determined. The vacuity contrast group received the same treatment. The content of EOC was calculated according to the difference between the sample and the vacuity contrast group.

### 2.5 Soil enzyme activities

Soil catalase activity was determined using the potassium permanganate titration (Guan et al., 1986; Li et al., 2014). 40 ml of distilled water and 5 ml of hydrogen peroxide solution (3%) were added to 2 g of soil, which was shaken for 30 min and then filtered. We then took 25 ml of the filtrate and titrated it to pink with 0.1 M potassium permanganate.

The urease activities were analyzed according to the methods used by Guan and Yin (Guan et al., 1986; Yin et al., 2014). The soil (2 g) was treated with 10 ml urea (10%), 20 ml citrate buffer (1 M, pH 6.7), and 1 ml of methylbenzene and stored at room temperature for 15 min. The sample was then shaken at 37°C for 24 hr. The solution was filtered, and 1 ml of the filtrate was mixed with 20 ml of distilled water, 4 ml of sodium phenolate hydroxide, and 3 ml of sodium hypochlorite. The NH_4_^+^‐N was analyzed 20 min later using a spectrophotometer at 578 nm. Urease activity was expressed in milligrams of NH_4_^+^‐N per gram of dry soil released in 24 hr.

The invertase activity and amylase enzyme activities were analyzed according to the methods used by Guan et al. (1986), and using 3,5‐dinitrosalicylic acid, the invertase activity and amylase activities were measured using sucrose solution and soluble starch as respective substrates, respectively. The invertase activity was expressed as the mass (mg) of glucose in 1 g of soil after 24 hr; the amylase activity was expressed as the mass (mg) of maltose in 1 g of soil after 24 hr.

### 2.6 Statistical analysis

We used the Tukey–Kramer method to analyze significant differences in the soil carbon component content and enzyme activity under different vegetation types. A two‐way ANOVA test was used to analyze the effects of vegetation type and soil layer depth on the SOC components and enzyme activities. The confidence interval was 95%, and *p* < .05 was considered significant. The errors in the figures and tables of this article are standard errors. The relationships between soil carbon fractions and soil properties (physical and chemical characteristics, and enzyme activities) were analyzed using a Pearson correlation analysis.

## 3 RESULTS

### 3.1 Variation of soil physical and chemical properties in different vegetation types

The vegetation types had significant effects on the soil's basic physical and chemical properties. The soil BD of the GL plot was higher than the other three vegetation types (Table 2), except for the 0–20 cm layer, the difference only between CK and GL reached a significant level. In the 0–20 cm layer, the TN and TP content of XS and HR soil was significantly higher than GL, while the soil TN content and TP content (*p* < .05) of CK were lower than GL plot. In the 20–40 cm layer, the soil TP of GL was significantly lower than that of the other vegetation types (*p* < .05); except for XS soil TP content was significantly lower than GL plot, the soil TN and TP content of other vegetation types were not significantly different from GL. The soil BD of the 20–40 cm layer under all four vegetation types was higher than that of 0–20 cm, while the total porosity, TN, and TP in the 20–40 cm were lower than the 0–20 cm layer.

**TABLE 2:** Basic properties of the soils for the four vegetation types

Vegetation	Soil layers (cm)	Bulk density (g/cm^3^)	Total porosity (%)	TN (g/kg)	TP (mg/kg)
XS	0–20	1.54 ± 0.02 AB	67.38 ± 1.67 A	**0.66 ± 0.00 A**	**43.15 ± 0.75 A**
	20–40	1.66 ± 0.02 A	56.17 ± 1.10 BC	0.33 ± 0.00 A	30.56 ± 0.97 B
HR	0–20	1.58 ± 0.02 A	62.46 ± 0.61 A	0.43 ± 0.00 B	42.13 ± 0.49 A
	20–40	1.65 ± 0.06 A	**66.41 ± 1.65 A**	0.37 ± 0.04 A	32.32 ± 1.00 AB
CK	0–20	1.50 ± 0.03 B	67.33 ± 1.01 A	0.39 ± 0.00 C	33.76 ± 0.12 C
	20–40	1.56 ± 0.08 A	63.00 ± 3.31 AB	0.31 ± 0.00 A	32.45 ± 0.36 AB
GL	0–20	**1.59 ± 0.02 A**	67.06 ± 2.43 A	0.40 ± 0.01 C	37.44 ± 0.48 B
	20–40	**1.71 ± 0.02 A**	54.59 ± 2.31 C	0.33 ± 0.01 A	34.63 ± 0.13 A

Note

Capital letters indicate that there are significant differences (*p* < .05) between vegetation types under the same soil layer (*n* = 12). The error is the standard error.

Abbreviations: CK, *Caragana korshinskii*; GL, Grassland; HR, *Hippophae rhamnoides*; TN, total nitrogen; TP, total phosphorus; XS, *Xanthoceras sorbifolia*.

### 3.2 Variation of soil carbon fractions in different vegetation types

**FIGURE 2:**
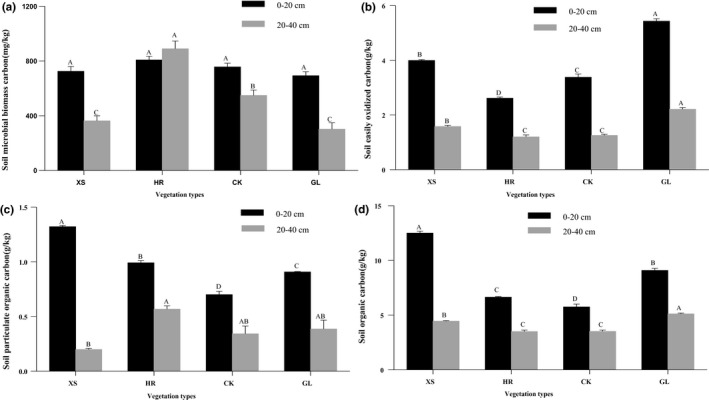
Vegetation type affects MBC (a; microbial biomass carbon), EOC (b; easily oxidized carbon), POC (c; particulate organic carbon), and SOC (d; soil organic carbon) content. CK, *Caragana korshinskii*; GL, Grassland; HR, *Hippophae rhamnoides*; XS, *Xanthoceras sorbifolia*. Different capital letters indicate significant differences among vegetation types for the same soil layer based on the Tukey–Kramer method at *p* < .05. The error bar is the standard error

There were significant differences in EOC, POC, and SOC under the four vegetation types (Figure 2). There were no significant differences among the four vegetation types of MBC content in the 0–20 cm layer, while soil MBC content in the GL was significantly lower than the other three vegetation types at the 20–40 cm layer. Except for the MBC content of the HR vegetation, the MBC, EOC, POC, and SOC content in other vegetation types decreased significantly as soil depth increased. At the 0–20 cm layer, the EOC content of the GL vegetation was 1.44, 2.82, and 2.06 g/kg higher than XS, HR, and CK, respectively. At the 20–40 cm layer, the EOC content of the GL was 0.63, 1.01, and 0.95 g/kg higher than XS, HR, and CK, respectively. Compared with GL treatment, the soil POC content of XS and HR plots in the 0–20 cm layer were significantly higher than that of GL, while the POC content of CK plots was significantly lower than GL (0.91 g/kg). The soil POC content of the 20–40 cm layer of the GL plot was not significantly different from the other three vegetation types. In addition, XS vegetation soil SOC content of the 0–20 cm was 37.47% higher than that of GL, while the HR and CK vegetation soil SOC content of 0–20 cm was 27.03% and 36.70% lower than GL (*p* < .05); GL vegetation soil SOC content of the 20–40 cm was 14.74%, 45.77%, and 45.56% higher than that of XS, HR, and CK, respectively. A two‐way ANOVA analysis demonstrated significant associations between soil depth and vegetation type on organic carbon components (MBC, EOC, POC, and SOC) in all the samples measured (Table 3).

**TABLE 3:** Two‐factor ANOVA analysis was used to test the differences in soil organic carbon components (microbial biomass carbon, MBC; easily oxidized carbon, EOC; particulate organic carbon, POC; soil organic carbon, SOC) and enzyme activities (amylase, catalase, urease, invertase). VT: vegetation type, SD: soil depth

	MBC			EOC			POC			SOC		
	*df*	*F*	*p*	*df*	*F*	*p*	*df*	*F*	*p*	*df*	*F*	*p*
VT	3	33.210	.000	3	298.827	.000	3	17.438	.000	3	313.404	.000
SD	1	65.499	.000	1	2,320.578	.000	1	449.429	.000	1	1837.468	.000
VT × SD	3	15.946	.000	3	61.852	.000	3	37.881	.000	3	159.691	.000

	Amylase			Catalase			Urease			Sucrase		
	*df*	*F*	*p*	*df*	*F*	*p*	*df*	*F*	*p*	*df*	*F*	*p*
VT	3	13.920	.000	3	1.260	.321	3	1,964.539	.000	3	107.105	.000
SD	1	93.711	.000	1	0.222	.644	1	10,051.341	.000	1	1,097.978	.000
VT × SD	3	7.227	.003	3	4.398	.019	3	712.897	.000	3	137.092	.000

### 3.3 Variation of soil enzyme activities in different vegetation types

**FIGURE 3:**
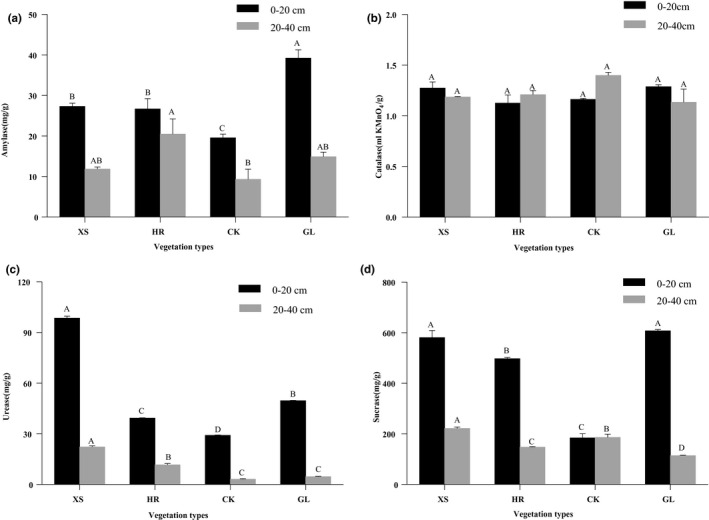
Vegetation type affects soil amylase (a), catalase (b), urease (c), and sucrase (d) activity. CK, *Caragana korshinskii*; GL, Grassland; HR, *Hippophae rhamnoides*; XS, *Xanthoceras sorbifolia*. Different capital letters indicate significant differences among vegetation types for the same soil layer based on the Tukey–Kramer method at *p* < .05. The error bar is the standard error

The vegetation types significantly affected soil amylase, urease, and sucrase activities (Figure 3). There were no significant differences in soil catalase activity among the four vegetation types. However, a two‐way ANOVA test revealed a significant association between soil depth and vegetation type on catalase activity (Table 3). For the 0–20 cm soil layer, the amylase activity in GL vegetation was significantly higher than that of the other three vegetation types (Figure 3a); the soil urease activity of XS plot was higher than GL by 48.85 mg/g, while HR and CK were lower than GL by 10.26 and 20.55 mg/g, respectively (*p* < .05) (Figure 3c); soil sucrase activity in GL vegetation was significantly higher than HR and CK by 110.23 and 423.35 mg/g, respectively, but no significant difference was observed in XS vegetation (Figure 3d). In the 20–40 cm soil layer, the difference between the soil amylase activity of the GL plot and other vegetation types did not reach a significant level, and the CK amylase activity was significantly lower than the HR plot. The soil urease activity in the 20–40 cm layer of XS and HR were significantly higher than GL by 17.64 and 7.05 mg/g, while the difference between CK and GL did not reach a significant difference. In the vertical section of soil, soil catalase of HR and CK showed upper layer < lower layer, while the other two vegetation types showed upper layer > lower layer. A two‐way ANOVA test demonstrated extremely significant relationships between soil depth and vegetation type on enzyme activity (amylase, urease, sucrase) in all samples studied (Table 3).

### 3.4 Relationships among the SOC fractions, enzyme activities, physical and chemical characteristics

A correlation analysis (Table 4) demonstrated that the MBC content displayed an extremely significant positive correlation with catalase. The POC was significantly correlated with urease, sucrase, TN, and TP; however, no significant correlations were observed with amylase, catalase, total porosity, and BD. The SOC was significantly correlated with urease, sucrase, TN, TP, total porosity, and BD. Soil sucrase activity displayed an extremely significant correlation with amylase and urease, with respective correlation coefficients of 0.597 and 0.848. Physical and chemical characteristics of the soil (i.e., TN, TP, total porosity, and BD) displayed strong positive correlation with urease and sucrase.

**TABLE 4:** The correlation coefficients between soil labile organic carbon and enzyme activities

	MBC	EOC	POC	SOC	Amylase	Catalase	Urease	Sucrase	TN	TP	Total porosity	Bulk density
MBC	1	−0.911**	0.596*	−0.136	0.206	0.694*	−0.197	−0.154	−0.117	−0.154	−0.058	−0.232
EOC		1	−0.378	0.459	−0.254	−0.485	0.487	0.347	0.423	0.456	0.369	0.517
POC			1	0.591*	0.492	0.518	0.582*	0.629*	0.629*	0.618*	0.549	0.525
SOC				1	0.069	0.317	0.984**	0.767**	0.990**	0.984**	0.954**	0.984**
Amylase					1	−0.258	0.198	0.597*	0.130	0.184	0.019	0.093
Catalase						1	0.197	−0.145	0.320	0.228	0.463	0.240
Urease							1	0.848**	0.980**	0.988**	0.925**	0.988**
Sucrase								1	0.776**	0.845**	0.632*	0.777**
TN									1	0.984**	0.952**	0.969**
TP										1	0.916**	0.969**
Total porosity											1	0.935**
Bulk density												1

Abbreviations: EOC, easily oxidized carbon; MBC, microbial biomass carbon; POC, particulate organic carbon; SOC, soil organic carbon; TN, total nitrogen; TP, total phosphorus.

* Significant relation at 0.05 levels.

** Significant relation at 0.01 levels.

## 4 DISCUSSION

### 4.1 Soil carbon fraction of different vegetation types

Vegetation is one of the most important components of an ecosystem, and its community succession has a significant effect on the SOC content (Deng et al., 2018; Solomon et al., 2007). This study demonstrated that SOC content in a forest was significantly higher than in shrublands and grasslands (Figure 2d). Root exudates and litter from forest vegetation both strongly affected the organic carbon content in the soil and promoted the effectiveness of forest nutrients (Qiao et al., 2014). At the same time, forest vegetation can also alter the forest environment, reducing solar radiation and temperature differences, increasing soil moisture (Özkan & Gökbulak, 2017), and creating a stable environment for litter decomposition. All of this causes the SOC content of forest to be higher than that of shrublands and grasslands. Moreover, due to the higher coverage of herbaceous vegetation and abundant species density (Table 1), more surface litter increases the sources of organic carbon (Zhang et al., 2019), making the SOC content of desert grassland vegetation higher than that of both HR and CK vegetation. Meanwhile, the SOC content of the four vegetation types was not only due to organic carbon inputs, but was also affected by enzyme activities and soil physical–chemical characteristics. A correlation analysis between SOC contents and soil physical–chemical characteristics and enzyme activities further confirmed these results (Table 4).

The MBC content in the soil of HR was significantly higher than in the soil of GL (Figure 2a). On the one hand, HR vegetation has a wide horizontal root structure and can quickly grow new shoots (Letchamo et al., 2018). These new shoots increase soil porosity (Table 2) and oxygen content during the growth process and increase soil aerobic microbial activity. On the other hand, the root nodules of HR can fix atmospheric nitrogen and improve soil fertility (their annual average nitrogen accumulation is 17,475 kg/hm^2^) (Ruan & Li, 2002). Studies have shown that increasing soil N can promote microbial activity and increase the decomposition rate of soil organic matter (Nottingham et al., 2012; Sistla et al., 2012), thereby reducing SOC content. The partial shading effect of XS vegetation reduces the soil temperature and the activity of soil microorganisms (Jiménez et al., 2007). Therefore, the soil MBC content is highest in HR vegetation.

The changes in soil POC and SOC are consistent (Figure 2c) across different vegetation types, while the changes in EOC and SOC differ (Figure 2b). Since the soil in this study was obtained from different vegetation types, different physical and chemical properties (Table 2) regulate the decomposition rates in the soil (Xu et al., 2016). Various surface litter can significantly change the input of soil organic matter (Thorburn et al., 2012), which affects the EOC content in the surface soil (DuPont et al., 2010). At the same time, the higher the soil temperature, the lower the soil water content, which may potentially create more beneficial conditions to enhance labile SOC fractions (Chen et al., 2016). However, the decomposition of plant litter is the most complex ecological process in the biosphere (Méndez et al., 2019). Therefore, due to the differences in tree species composition, litter quantity and quality, soil microbial group composition, soil moisture, temperature, and nutrient input, different vegetation types have significant differences in soil active organic carbon components (Soucémarianadin et al., 2018; Yang et al., 2018).

The content of activated carbon in the soil under the four vegetation types was greater in the upper layer than in the lower layer. This was mainly because the soil active organic carbon largely depends on the total organic carbon content of the soil. Total SOC decreased as soil depth increased (Figure 2d), however, the litter on the upper layer not only provides a significant amount of organic carbon for the soil, but also provides the surface soil with a high concentration of nutrients (Table 2), providing stable conditions for growing fine roots in the topsoil layer. Litter and root exudates have become an important source of soil active organic carbon after they are decomposed by microorganisms (Weintraub et al., 2007).

### 4.2 Soil enzyme activity of different vegetation types

Our study shows that different vegetation types affect soil enzyme activity differently (Figure 3). Urease, a key enzyme that regulates soil nitrogen transformation, comes mainly from plants and microbes and plays a key role in nutrient cycling (Zhao et al., 2012). Soil urease activity in XS vegetation is higher than in the others (Figure 2c). The high urease activities in XS vegetation may be due to both microbial growth and stimulation of microbial activity by enhanced resource availability (Li et al., 2014). At the same time, higher soil nutrients (Table 2) and SOC contents (Figure 2d) provide microorganisms with a rich source of nitrogen and carbon, which significantly adds to the nutrients accumulated by transformation (Cui et al., 2019). Improving the physical properties of soil creates an environment that benefits microorganisms (Iovieno et al., 2009) and increases urease activity.

Catalase can decompose hydrogen peroxide into molecular oxygen and water to prevent cells from being damaged by reactive oxygen species (Bartkowiak & Lemanowicz, 2017). In this study, we found no significant difference in soil catalase activity under different vegetation types. This may be due to less rainfall in this area, and small differences in soil microbial activity (MBC, Figure 2) and soil properties (bulk density and porosity, Table 2), leading to there was no significant difference in soil catalase activity. Furthermore, microbial communities, litter decomposition, and soil pH are also important factors affecting soil catalase activity (Brzezińska et al., 2005; Gu et al., 2009; Kannan & Wei, 2008).

Our research results show that forest soil has the highest activity of urease and sucrase. This may be due to the fact that more root exudates in forest increase soil nutritional content (Canarini et al., 2019); that is, higher soil nutrient content (SOC, TN, TP) provides sufficient energy for microbial activities. At the same time, the physiological metabolism process of forest roots will release more kinds of enzymes into the soil (Nardi et al., 2002). Moreover, a large number of animals in forest land will also contribute to the improvement of soil enzyme activity (Liu et al., 2019). The higher activity of amylase and sucrase in the surface soil (0–20 cm) of the GL plot, which is because there are more types of vegetation and litter on the surface of GL, the higher content of SOC fractions (Figure 2), and high input capacity of soil organic matter affects the community structure and growth of rhizosphere soil microorganisms (Prescott, 2010). At the same time, GL is dominated by low herbaceous plants (Table 1). The shadow effect of this vegetation type is small, and the soil temperature is higher than the other three vegetation types, resulting in higher soil amylase and sucrase activities of GL vegetation. In addition, the soil amylase activity in the 20–40 cm layer of the HR plot is higher. This may be the higher MBC, POC content (Figure 2a,c) and total porosity (Table 2) in the 20–40 cm layer of the HR plot provide higher oxygen and substrates for the microorganisms, while the roots of GL vegetation are mainly concentrated in the 0–20 cm layer (An et al., 2009), which means that the amylase in HR vegetation is more active in the 20–40 cm layer.

In all four vegetation types, the soil amylase, urease, and sucrase activities were greater in the upper layer than in the lower layer, while the soil catalase activity did not change significantly. Due to the high SOC content (Figure 2), there are sufficient nutrient sources to facilitate the growth of microorganisms. In addition, higher surface temperatures and better ventilation enable soil microorganisms to quickly grow and metabolize (Chen et al., 2019). The underground biomass in the 20–40 cm soil layer was reduced, which reduces the source of soil nutrients, while this reduction of SOC content and plant roots often leads to a decrease in enzyme activity (Xiao et al., 2015). These results suggest that the effects of vegetation on soil enzyme activities are different under different soil types and environmental conditions. Moreover, our research also showed that vegetation types and soil depth have significant interactions on carbon components and enzyme activities. This may be because after vegetation restoration, litter and root exudates mainly affect surface soil microorganisms and soil nutrients, while this effect will be significantly weakened in deeper soil layers (Eilers et al., 2012; Xu et al., 2020). At the same time, vegetation restoration protects enzyme substrates by improving soil structure, and promotes the contact of soil enzymes with SOC, resulting in increased soil enzyme activity (Allison & Jastrow, 2006). The increase of soil depth leads to poor soil ventilation, and microorganism's energy demand, enzyme substrates, and soil activated carbon content gradually decrease (Stone et al., 2014).

### 4.3 Relationship between soil carbon fraction and enzyme activity

Enzymes participate in the transformation process of soil nutrients. Enzyme activity plays a vital role in soil microbial activity and soil quality (Ebhin Masto et al., 2006). Under stable organic nutrient conditions, soil enzyme activity is typically higher, and increased mineralization of the soil's nutrients creates a more favorable environment for nutrient cycling (Roldán et al., 2005). The results of this study demonstrate that catalase activity was significantly related to MBC content, and can reflect the changing process of MBC. Both urease activity and sucrase activity displayed significant positive correlations with organic carbon and total N content. Urease and sucrase activity can reflect the decomposition of organic matter and nitrogen in soil and can be used as important indicators of soil fertility. In sum, enzyme activity and carbon fraction influence each other's conversion and circulation of nutrients (Qi et al., 2016; S. Zhao et al., 2016).

## 5 CONCLUSIONS

This study analyzed the responses of soil organic carbon fractions and related enzyme activities to different vegetation types in the northern Loess Plateau. Our results demonstrated that vegetation types significantly affected the distribution characteristics of soil active organic carbon components (SOC, MBC, EOC, POC) and enzyme activities (amylase, urease, sucrase). Among the four vegetation types, the forest (XS) had a significantly higher SOC content, urease, and sucrase activity, the grassland (GL) had a significantly higher EOC content and amylase activity, and the shrubs had a significantly higher MBC content. In the soil profile, the content of soil SOC, EOC, POC, and activities of soil amylase, urease, and sucrase were all significantly higher in the upper than in the lower layer. The results suggest that forest systems are more conducive to SOC storage due to stable environmental factors. Moreover, we also found that the MBC significantly impacted catalase activities, POC significantly impacted urease and sucrase activities, and SOC significantly impacted urease and sucrase activities. This is because the availability of soil carbon partially controls the activity of microorganisms and enzymes; that is, changes in soil enzyme activities would depend on the way of expressing activity. Overall, this study provides valuable information on the distribution of SOC fractions and enzyme activities of the four vegetation types in the Loess Plateau that may contribute to our understanding of SOC sequestration. Considering the effects of vegetation on litter decomposition and soil microbial community structure will jointly affect the soil carbon dynamics, more long‐term research is needed to better understand the dynamic mechanism of soil organic carbon fractions after returning farmland to forest.

## CONFLICT OF INTEREST

The authors declare no conflict of interest.

## AUTHOR CONTRIBUTIONS

**Haiyan Wang:** Data curation (equal); investigation (equal); writing – original draft (equal); writing – review and editing (equal). **Jiangqi Wu:** Conceptualization (equal); data curation (equal); investigation (equal); methodology (equal); resources (equal); software (equal); validation (equal); writing – original draft (equal); writing – review and editing (lead). **Guang Li:** Funding acquisition (equal); project administration (equal). **Lijuan Yan:** Funding acquisition (equal); project administration (equal).

## Data Availability

The soil organic carbon fractions and enzyme activities’ data are available in Dryad: https://doi.org/10.5061/dryad.jwstqjq68.
